# Engineering biomaterials by inkjet printing of hydrogels with functional particulates

**DOI:** 10.1007/s44258-024-00024-4

**Published:** 2024-07-03

**Authors:** Cih Cheng, Eric J Williamson, George T.-C. Chiu, Bumsoo Han

**Affiliations:** 1 School of Mechanical Engineering, Purdue University, West Lafayette, IN USA; 2 Purdue Institute for Cancer Research, Purdue University, West Lafayette, IN USA; 3 Department of Mechanical Science and Engineering, Materials Research Laboratory and Cancer Center at Illinois, University of Illinois Urbana-Champaign, 1206 W Green St, Urbana, IL 61801 USA

**Keywords:** Drop-on-demand printing, Water-matrix interaction, Particle transport, Tissue engineering, Drug delivery device, Bioelectronics

## Abstract

Hydrogels with particulates, including proteins, drugs, nanoparticles, and cells, enable the development of new and innovative biomaterials. Precise control of the spatial distribution of these particulates is crucial to produce advanced biomaterials. Thus, there is a high demand for manufacturing methods for particle-laden hydrogels. In this context, 3D printing of hydrogels is emerging as a promising method to create numerous innovative biomaterials. Among the 3D printing methods, inkjet printing, so-called drop-on-demand (DOD) printing, stands out for its ability to construct biomaterials with superior spatial resolutions. However, its printing processes are still designed by trial and error due to a limited understanding of the ink behavior during the printing processes. This review discusses the current understanding of transport processes and hydrogel behaviors during inkjet printing for particulate-laden hydrogels. Specifically, we review the transport processes of water and particulates within hydrogel during ink formulation, jetting, and curing. Additionally, we examine current inkjet printing applications in fabricating engineered tissues, drug delivery devices, and advanced bioelectronics components. Finally, the challenges and opportunities for next-generation inkjet printing are also discussed.

**Graphical Abstract:**

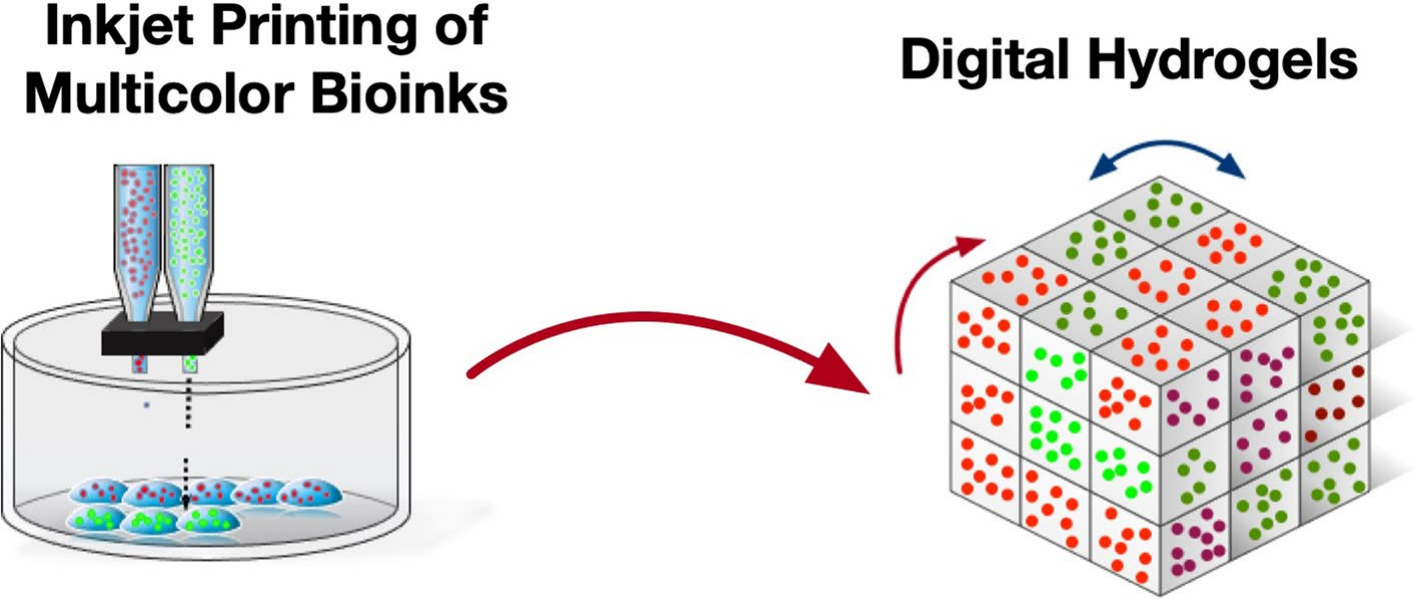

## Highlights

• Inkjet printing recently emerged to construct hydrogels with particulates into advanced biomaterials and biomedical devices.

• A fundamental understanding of intra and inter-drop transport processes begins to be established.

• Challenges and opportunities for next-generation inkjet printing are also discussed.

## Introduction

Recent advances in polymer sciences have led to the development of novel hydrogels with customizable properties, paving the way for various emerging applications. These applications include tissue engineering [1], drug delivery [2], stretchable and wearable electronics [3], and energy storage and conversion [4]. Incorporating particulates, such as proteins, drugs, nanoparticles, and cells, into hydrogels has facilitated the creation of innovative biomaterials with enhanced mechanical, biological, electrical, and chemical properties.

To scale up these laboratory-level materials to practical products, there is a pressing need for manufacturing methods capable of fabricating three-dimensional (3D) hydrogel-based products with tailored functional properties. Consequently, many techniques have been developed to create hydrogel-based soft biomaterials. One innovative method is electro-addressing, which utilizes electric fields to manipulate and position cells and particles within hydrogels to align them in desired orientations [5, 6]. However, electro-addressing also faces challenges, such as the requirement for conductive hydrogel formulations and the potential effects of electric fields on cell viability and function. Among these techniques, 3D printing has rapidly emerged, as it can directly produce hydrogel products with complex geometries through layer-by-layer deposition [7]. Moreover, the printing of particulate-laden hydrogels has gained popularity due to the growing interest in drug delivery devices with tailored release profiles, personalized implants and tissue models, and specific surgical tools for unique cases [8–10]. Efforts in material science, multiscale simulations, manufacturing methods, and design engineering have collectively enabled these promising scientific ends.

The three main categories of 3D printing are extrusion-based, laser-assisted, and jetting-based printing [11, 12]. While extrusion-based printing offers affordability and versatility with thermoplastic materials, it typically yields lower resolution with a range of 100 to 1000 μm [13] and requires additional finishing for surface smoothness. Laser-assisted printing is capable of producing parts with good mechanical properties at high precision with surface features between 10 and 100 μm [13]. However, the process is slower, more expensive, and involves the generation of heat, which can be detrimental to sensitive materials or biological components, with recent work showing a temporary alteration of cell phenotype [14, 15]. Inkjet printing, on the other hand, provides high accuracy with feature sizes between 20 to 100 μm and is suitable for printing multiple materials and colors simultaneously [13]. However, it is limited to materials that can be jetted and cured [16].

**Fig. 1:**
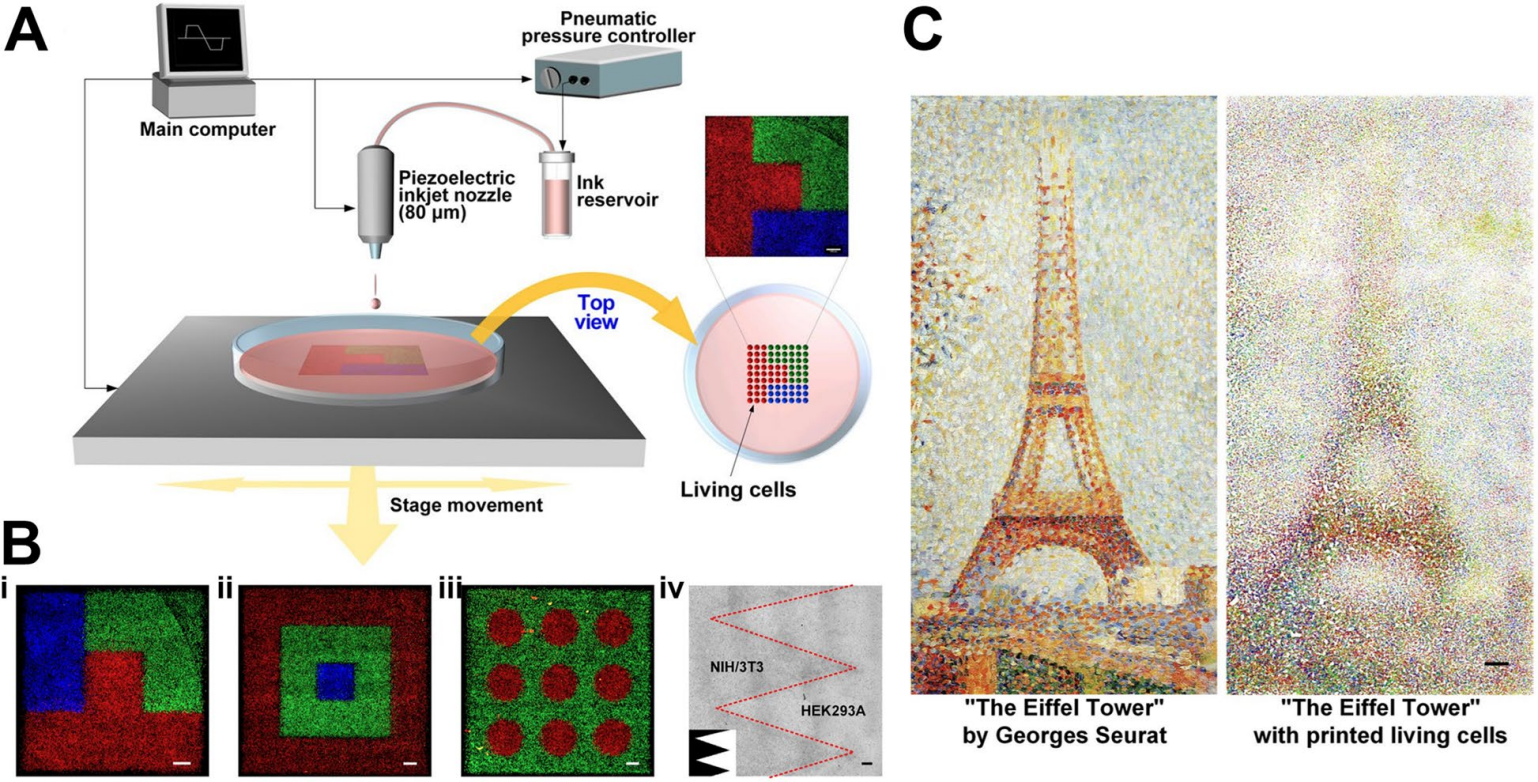
**A** Schematic of the direct inkjet printing setup. **B** Demonstration of various cell patterns that were directly printed into culture medium: (i-iii) Fluorescent images of red-, green-, and blue-labeled cell patterns; (iv) printed zigzag pattern using a heterotypic coculture model of NIH3T3 and HEK293A cells. **C** “Te Eifel Tower” by Georges Seurat was patterned with cells labeled with 3 colored dyes by direct inkjet printing. Adapted from [19] under a Creative Commons license

Despite these advancements, most current manufacturing methods of hydrogels struggle to produce advanced hydrogels with spatially varying compositions and properties [17]. The development of techniques capable of creating hydrogels with such spatial variations holds the promise of next-generation "digital hydrogels." Among all the methods, inkjet printing, also known as drop-on-demand (DOD) printing, shows great potential for addressing these manufacturing challenges [18]. DOD printing can construct 3D parts by additively depositing drops of multiple polymer inks at desired locations with appropriate timing between adjacent drops. Thus, DOD printing enables the construction of 3D hydrogel configurations with precisely controlled properties and compositions. This superior spatial controllability of DOD printing complements widely used extrusion-based printing methods. As seen in Fig. 1, the controllable cell patterns were demonstrated by direct inkjet printing [19]. Depending on ink and nozzle properties, back pressure may be required to prevent ink from falling uncontrolled out of the nozzle. Precise pressure control is required to ensure droplet formation can continue at the nozzle tip; a mechanism for this purpose is shown in Fig. 1A. If the droplet does not form at all or forms too large, printing resolution may be impacted, and the process could entirely fail due to no liquid being ejected.

Collectively, 3D printing of hydrogel has emerged as a transformative technology that offers unprecedented opportunities to design and fabricate complex structures tailored to individual needs. Among the various printing techniques, inkjet printing stands out due to its potential to achieve precise particle distribution within hydrogels. This precision is pivotal in the creation of particulate-laden hydrogels, which have significant applications in implants, drug-delivery devices, and biological tissues. The ability to control the spatial distribution of particles, be it cells, biomolecules, or drugs opens the door to fabricating biological tissues that mimic native structures and functions. Furthermore, this technology holds the promise of revolutionizing drug delivery by enabling devices with customized release profiles, ensuring optimal therapeutic outcomes. This review aims to explore the advancements and challenges of inkjet printing of hydrogels, with a focus on its applications in biological tissues, drug delivery systems, and bioelectronics.

## Inkjet printing of hydrogel-based biomaterials

### Hydrogels

Hydrogels are highly hydrophilic networks of polymer chains utilized in many biomedical applications, including biosensors [20], cell encapsulation [21], and drug delivery [22]. Their ability to absorb a large amount of water, effective mass transfer, similarity to natural tissues, and the ability to form various shapes, make them ideal materials to pattern cells in three-dimensional (3D) configurations using 3D printing.

Both natural and synthetic hydrogels are broadly employed. The common natural polymers exploited for the fabrication of hydrogels are biodegradable materials such as fibrin, collagen, hyaluronic acid (HA), and alginate that can mimic natural tissue constructs. On the other hand, synthetic hydrogels such as poly(ethylene glycol) (PEG), poly(acrylamide) (PAAM) and poly(vinyl alcohol) (PVA) possess controllable chemical and mechanical features. However, synthetic hydrogels must be modified for biocompatible by associating with other molecules upon polymerization. Natural hydrogels are viscoelastic materials more suitable for engineering soft tissue, whereas using synthesized derivatives to create synthetic hydrogels can achieve structures with more customizable mechanical properties.

Due to their superior softness, responsiveness, and biocompatibility, hydrogels are being intensively investigated for use in stretchable devices and machines, including sensors, actuators, electronics, and water harvesters. Their optimized swelling/shrinkage properties make them suitable as sensors for converting external stimuli into electromechanical signals. Studies are attempting to enhance the electrical conductivity of hydrogels, achieving various hydrogel electronics, as will be discussed later.

Overall, hydrogels are highly biocompatible systems capable of encapsulating cells and biomolecules uniformly. They possess good porosity for oxygen, nutrient, and metabolite diffusion, and can be processed under mild cell-friendly conditions, making them versatile biomaterials for tissue engineering, drug discovery, and biological research.

### Ink formulation

**Fig. 2:**
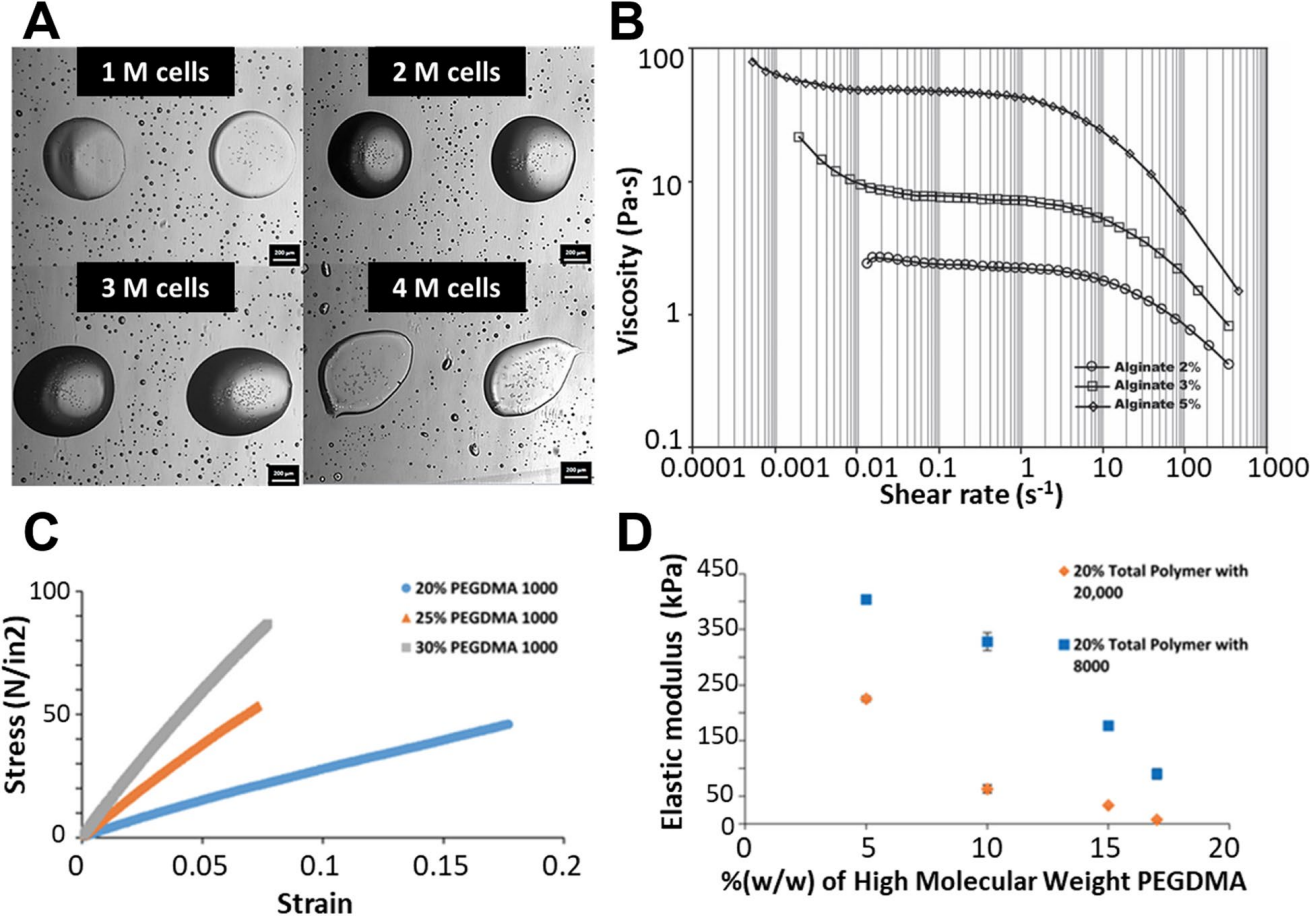
**A** Representative images of deposited droplets on well plates; the increased probability of nozzle clogging occurs at higher concentrations. Adapted from [23] with permission from the Royal Society of Chemistry. **B** Viscosity variation as a function of shear rate for different alginate solutions. Shear thinning is demonstrated by the rapid decline in viscosity as the shear rate is increased, with higher concentration alginate having the greatest reduction in shear viscosity. Adapted from [24] with permission from Wiley–VCH. **C** Effects of varying the total PEGDMA 1000 percentage on hydrogel elasticity. A decrease in the total polymer percentage resulted in a decreased elastic modulus. **D** An increase in the percent wt. of high molecular weight PEGDMA into the PEGDMA 1000 hydrogels resulted in a decreased elastic modulus. The composition of each hydrogel includes the given percentage of higher molecular weight (Mw) polymer with the remainder of the hydrogel composed of PEGDMA 1000. Reprinted with permission from [30]. Copyright 2017 American Chemical Society

In the biomedical area, the "ink" is often a bio-ink or a biomaterial ink. Bioinks contain living cells and biomaterials resembling the extracellular matrix environment, supporting cell adhesion, proliferation, and differentiation post-printing. Biomaterial inks refer to a broader category of printing materials that may or may not contain living cells. These inks are used to create structures for biomedical applications, including scaffolds for tissue engineering, drug delivery systems, and medical devices. The viscosity, surface tension, and other rheological properties of the ink are crucial for successful printing [23–25]. The ink should have an optimal viscosity allowing smooth flow through the printer nozzle without clogging yet be thick enough to maintain structure post-printing [23, 26]. The addition of rheological modifiers, crosslinking agents, and cell suspensions can alter these properties [25, 27]. For example, a study has shown that increasing cell concentration in a bioink may reduce printing precision and increase nozzle clogging, as shown in Fig. 2A. In addition, shear-thinning behavior in inks is advantageous as it aids in easy flow under shear stress (during printing) and regains viscosity once the stress is removed, aiding structure retention [26, 28]. Sodium alginate is a polymer exhibiting strong shear-thinning behavior, as seen in Fig. 2B. Achieving an optimal balance is essential for successful printing, as it affects the droplet formation, size, and overall resolution of the printed structure. Because of the layer-by-layer process, each droplet/layer must provide the structural integrity for holding the subsequently printed drop/layer. Thus, the gelation mechanism is essential to transform a liquid ink into a solid (gelled) material. This is highly related to the degree of gelation/cross-linking achieved, which will be highlighted in the below sections.

In addition to the printing-related considerations, matching hydrogel properties with the target tissue is also critical. A hydrogel's structural and mechanical properties must align with desired functionalities [24], considering that tissue has a wide range of mechanical properties, from soft tissues such as fat, skin, and muscle, to hard tissues such as cartilage and bone [29]. As shown in Fig. 2C and D, a study has developed a hydrogel-based platform with tunable mechanical properties [30].

In bioinks containing cells, the biological properties of the hydrogel, such as biocompatibility, biodegradability, and biomimicry are essential to ensure cell viability, proliferation, and differentiation [31–33]. Incorporating bioactive molecules, such as growth factors, cytokines, or other signaling agents [34], requires a thorough examination of interactions or reactions between hydrogel and biomolecules. Factors like biodegradation, swelling, chemical binding, and physical entrapment can significantly impact the properties of the ink [25, 33]. These, consequently, influence the printability as well as the performance and functionality of hydrogel-based systems.

### Jetting

Inkjet printing is a non-contact digital printing technology that expels droplets of ink onto a substrate in a controlled manner. Two primary methods for droplet formulation include thermal inkjet and piezoelectric inkjet [16, 35]. In a thermal inkjet, a resistive heater located near the nozzle rapidly heats a small volume of ink upon the application of an electric pulse, causing it to vaporize and expand, forming a droplet at the nozzle tip. A piezoelectric inkjet printer consists of a piezoelectric piston that changes the position (displacement) when an electric pulse is applied, compressing the printing nozzle and forcing a droplet of ink out. The ejected droplets travel a short distance in the air and land on the substrate (e.g., paper, glass slide, etc.). The precision of the printer determines the exact placement of each droplet. The design of the printhead nozzle impacts the jetting behavior. Nozzle diameter, shape, and surface properties must be tailored to the specific properties of the hydrogel ink. Smaller nozzles allow for finer resolution but may increase the risk of clogging, especially with inks containing cells or larger particles. The printing parameters, such as the droplet ejection frequency and the velocity, need to be finely tuned to ensure consistent droplet formation and to prevent issues like satellite droplet formation or nozzle drying [16].

The jetting behavior of ink is determined by its elasticity, viscosity, and surface tension [36]. The Ohnesorge number, a dimensionless parameter, is commonly used to predict the jetting ability of Newtonian fluids, defined as follows (Eq. 1: Definition of Ohnesorge Number).

1 $$Oh\mathit=\frac{\mathit\mu}{\sqrt{\mathit\rho\mathit\sigma\mathit L}}$$

Here, $\mu$ represents the dynamic viscosity of the printing fluid, $\rho$ is its density, and $\sigma$ is the fluid surface tension. *L* denotes the characteristic length, usually the diameter of a droplet formed during the jetting process. This number encapsulates the interplay between inertial, viscous, and surface tension forces of the fluid [37]. To allow successful jetting, the Ohnesorge number of the fluid in the jetting environment must fall between 0.1 and 1.0. While material properties such as density and surface tension are relatively fixed for a given material, the viscosity of non-Newtonian materials may change depending on external factors. Hydrogels mostly exhibit non-Newtonian behavior such as a shear-rate-dependent viscosity and viscoelasticity. These properties directly influence the jetting process, affecting droplet formation, stability, and overall printing precision. The intricate relationship between the Ohnesorge number and the rheological characteristics of hydrogels necessitates careful formulation adjustments to optimize printing performance [37]. In the case of inkjet printing particle suspensions, the continuum assumption breaks down as the diameter of the liquid thread decreases and becomes comparable to the size of the particles. Therefore, particles may be trapped within the liquid thread, leading to the formation of “satellite drops”, where smaller fluid droplets are deposited on the substrate outside of the desired position [38]. Additionally, the use of particle suspensions can introduce a settling issue in which the particles may begin to agglomerate and lead to an altered flow of the ink through the nozzle. Direct imaging of the jetting process has improved the inkjet printing of particulate-laden hydrogels greatly as it allows for an improved understanding of droplet behavior during flight and adjustments to printing parameters to be made accordingly [39].

**Fig. 3:**
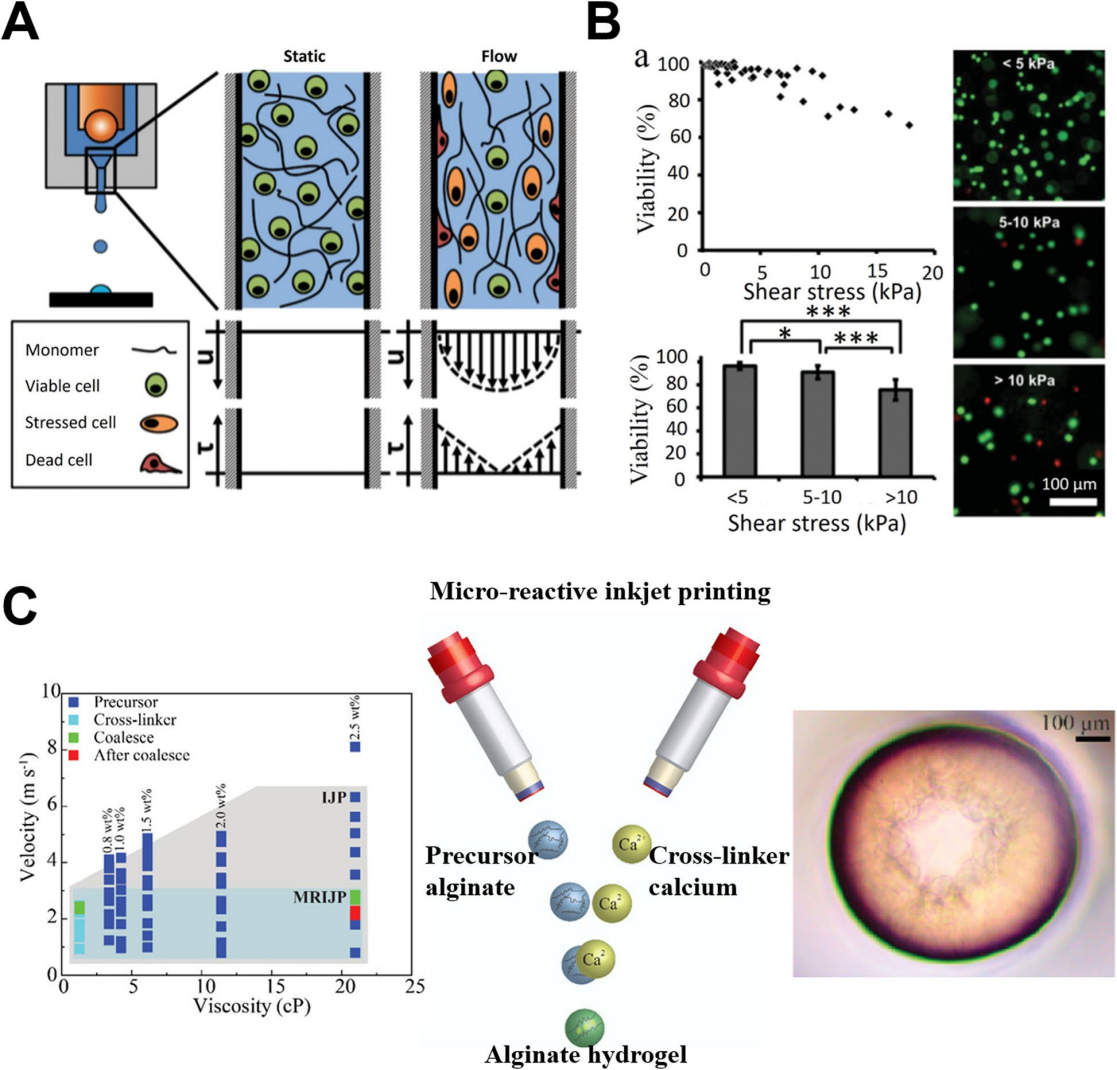
**A** Schematics for the distribution of velocity (u) and shear stress ($\uptau$) of the cell-laden hydrogels within a nozzle. **B** Effect of shear stress on cell viability after printing. The microscopic images on the right showed the live (stained in green)/dead (stained in red) cells after printing. Modified from [41] with permission from Elsevier under a Creative Commons license. **C** Microreactive inkjet printing by spontaneous inkjet printing of hydrogel precursor and cross-linker. Reprinted with permission from [42]. Copyright 2020 American Chemical Society

Jetting complex hydrogels, especially those containing living cells or multiple components, presents additional challenges [36]. Ensuring cell viability and uniform distribution within the printed structure is critical [35]. The shear forces experienced during jetting can impact cell health. The simplified illustrations of the shear stress before and during jetting are shown in Fig. 3A. It is noted that the real shear stress has a dramatic variation around the nozzle orifice mainly during the jetting impulse and the following dwell time. After that, the backflow fluid will push the fluid flowing down along the wall, increasing the wall shear stress simultaneously. The impulse strength and nozzle diameter have important effects on shear stress [40]. If these considerations are not taken, cell viability may be impacted owing to increased shear stress as seen in Fig. 3B. The printing parameters, such as printing pressure, diameter of the nozzle, and viscosity of the ink will determine the shear stress exerted on the bioink and the embedded cells [41]. Additionally, the presence of cells or other bioactive agents can affect the rheological properties of the ink, requiring a dynamic approach to ink formulation and printing parameter adjustment. One approach is utilizing reactive inkjet printing [42], where a precursor and cross-linker are printed simultaneously and form a hydrogel before deposition, as shown in Fig. 3C. The in-air coalescence of the precursor and cross-linker enabled single-step printing with zero influence on the printability and deposited pattern.

### Curing

Curing solidifies the printed material, transforming it from a liquid or gel state into a stable, solid form. It ensures that the printed structure maintains its shape and mechanical properties. In biomedical applications, curing is essential to ensure that the final product is biocompatible and safe for its intended use.

Some commonly used curing methods include UV curing [43], thermal curing [44], and chemical curing [45]. In biomedical applications, material sensitivity is one of the challenges in curing steps. Biomaterials, especially those containing living cells or sensitive proteins, can be affected by the curing process. It's crucial to choose a method that doesn't harm these components. Achieving uniform curing throughout the structure can also be challenging, especially for thicker or more complex geometries.

To optimize the curing step during inkjet printing, tuning various parameters is essential. This includes adjusting the curing time and intensity, ensuring it's neither too short to leave the material uncured nor too long to degrade it. In thermal curing, the temperature must be precisely controlled to avoid thermal degradation while effectively curing the material [46]. For chemical curing, the concentration of the curing agent is critical, as an imbalance can lead to inadequate curing or alter the material's properties [47]. Different biomaterials have unique responses to curing processes. Understanding the specific requirements of each material is key. In bioprinting, for instance, maintaining cell viability during and after the curing process is crucial. The curing conditions must be optimized to ensure that the cells remain alive and functional, without compromising the material's integrity. Sometimes, additional post-curing treatments like washing, drying, or the addition of bioreactors are required to achieve the desired material properties and functions [48]. After curing, assessing the mechanical, chemical, and biological properties of the material can provide valuable feedback for further optimization.

The curing process must be seamlessly integrated with the printing process, especially in continuous printing operations. Implementing real-time monitoring and control systems can be beneficial in adjusting curing parameters on-the-fly to achieve optimal results [36]. Otherwise, the water-matrix, drop-drop, or layer-layer interactions should be further studied to ensure product integrity since a complex fluid-matrix interaction occurs during curing. These interactions become even more complicated when particulates are added to the hydrogels. However, the fundamental mechanism is poorly understood. DOD printing processes of hydrogel-based inks have primarily been developed through time-consuming and labor-intensive trial-and-error experiments.

**Fig. 4:**
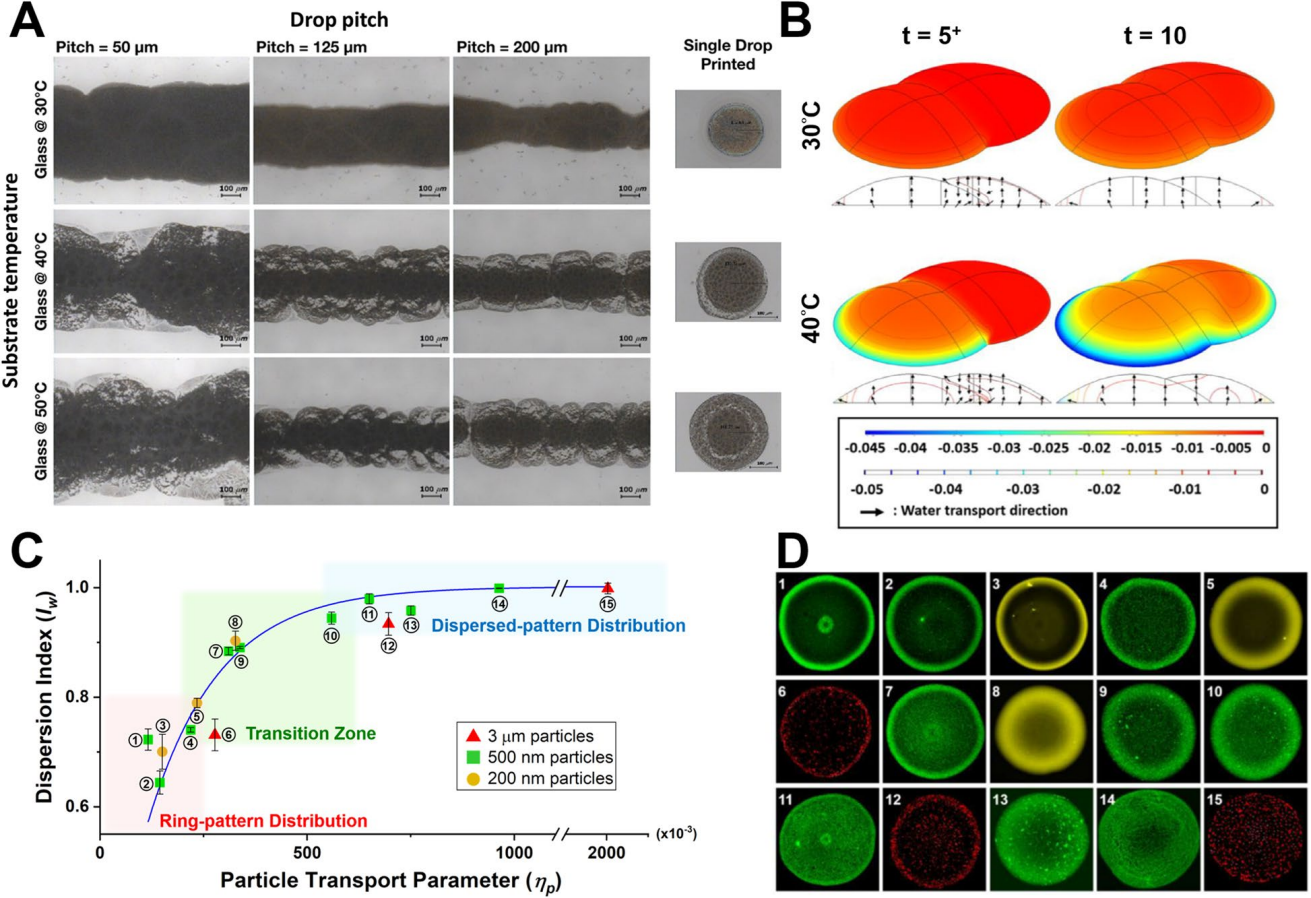
**A** Experimental results showing the effect of printing pitch and substrate temperature on adjacent dropwise gelation of printed drops. Reprinted from [50] with permission from Elsevier; **B** Computational dilatation results showing the effect of curing temperature on adjacent dropwise gelation of printed drops. Reprinted from [50] with permission from Elsevier; **C** Correlation between experimental particle distribution results and particle transport parameter following the same curve, and **D** the representative fluorescence photographs of particle distribution for each experimental case. Modified from [51] with permission from Elsevier

Previously, dehydration of a DOD-printed polymer drop and the interaction mechanism between the polymer matrix and interstitial water during dehydration have been proposed to predict the desired microstructure of printed hydrogels by a dimensionless parameter $\eta$, defined as below [49] (Eq. 2: Definition of water transport parameter).

2 $$\eta =\frac{\overline{{h }_{m}}}{{D}_{con}/{L}_{c}}\sim \frac{Water\ evaporation}{Interstitial\ water\ transport}$$

where ${L}_{c}$ is the characteristic length of the drop (the diameter of the drop or pitch distance between drops), ${D}_{con}$ is the consolidation constant of the polymer, and $\overline{{h }_{m}}$ is the average mass transfer coefficient at the drop-air interface. This dimensionless parameter represents the significance of water evaporation concerning to interstitial water transport. As $\overline{{h }_{m}}$ or ${L}_{c}$ increases or the ${D}_{con}$ decreases, the parameter will increase, which suggests rapid evaporation or slow interstitial water transport. The drop will be subjected to highly localized shrinkage. On the contrary, as $\overline{{h }_{m}}$ or ${L}_{c}$ decreases or the ${D}_{con}$ increases, the parameter implies that the interstitial water transport occurs rapidly so that the drop will experience less of a deformation gradient and spatially uniform matrix microstructure. Moreover, multi-drop dehydration that considers drop-drop interaction during curing based on the above theoretical model was further studied. Both experiments and simulations were performed to establish the mechanism of the water-matrix interaction during the curing of hydrogel drops [50], as shown in Fig. 4A, B.

Additionally, when the particulates are added, the transport processes of water and particulates within hydrogel during curing are also studied [51]. In this study, a dimensionless similarity parameter was introduced, determined by the particle size, hydrogel’s water evaporation coefficient, and mechanical properties, defined below (Eq. 3: Definition of particle transport parameter.

3 $${\eta }_{p}=\frac{\overline{{h }_{m}}}{\frac{{D}_{con}}{{L}_{c}}/\frac{{d}_{p}}{{L}_{c}}}=\frac{\overline{{h }_{m}}}{{D}_{con}/{d}_{p}}\sim \frac{Water\ evaporation}{Interstitial\ particle\ transport}$$

This is by assuming interstitial particle transport is proportional to the interstitial water transport. The parameter represents the significance of the water evaporation with respect to interstitial particle transport. Thus, it can be used as a quantitative indicator of particle movement during the curing and a predictor of the resulting intra-drop distribution of particulates after curing. The results confirmed a scaling law capable of guiding ink formulation and curing protocol as demonstrated in Fig. 4C, D.

Furthermore, curing soft biomaterials for inkjet printing, particularly when fabricating complex 3D structures, presents significant challenges due to the inherent mechanical instability and fluid dynamics of such materials. One new approach deposits ink directly into a support bath as opposed to a traditional printing platform. This helps prevent constructs from settling and collapsing [52].

In summary, the optimization of the curing step in inkjet printing of biomaterials is a complex and multifaceted process. It involves a careful balance of curing parameters, material compatibility, environmental conditions, post-curing treatments, and integration with the overall printing operation [53]. This optimization is crucial for achieving the desired material properties and ensuring the success of the final printed product, particularly in sensitive applications like the 3D printing of hydrogel.

## Emerging biomedical advances

### Inkjet printing of hydrogel in tissue engineering

Tissue engineering has long been at the forefront of regenerative medicine, aiming to restore, maintain, or enhance tissue and organ functions. This is a multidisciplinary field that seeks to develop biomimetic structures, replicating the intricate architecture and function of native tissues [54]. Hydrogels, with their high water content, biocompatibility, and tunable mechanical properties, have emerged as a choice material for such scaffolds due to their structural similarity to the native ECM and their ability to support cell growth and differentiation [53]. Recent advancements in hydrogel inks have significantly enhanced the capabilities of inkjet printing in tissue engineering. Innovations in ink formulations, such as the incorporation of natural polymers or synthetic hydrogels, have improved printability and biocompatibility [33, 55]. The development of inks with self-healing properties or temperature-responsive behavior has also opened new avenues for creating more complex and functional tissue constructs [56].

The true potential of hydrogels in tissue engineering is being realized through the integration of advanced manufacturing techniques, notably inkjet printing. When applying inkjet printing of hydrogels in tissue engineering, allows for the precise patterning of cells, growth factors, and other bioactive agents, creating 3D constructs that closely mimic the spatial heterogeneity of natural tissues [57]. This precision is particularly crucial in engineering complex tissues, where cellular arrangement, matrix composition, and mechanical properties need to be meticulously orchestrated. The integration of inkjet printing and hydrogels in tissue engineering has opened new avenues for research and innovation. From creating vascularized tissues [58] to multi-material constructs with gradient properties [59], the possibilities are broad and promising. Furthermore, with the continuous evolution of both hydrogel formulations and printing techniques, there is an ever-growing potential to address some of the most pressing challenges in regenerative medicine.

Inkjet printing technology has shown significant promise in the fabrication of organoids and spheroids, which are the most basic tissue models in tissue engineering. The ability of inkjet printing to precisely deposit cells and biomaterials layer by layer allows for the creation of complex, three-dimensional structures that closely mimic the architecture of natural organs [60]. This precision is crucial for developing organoids and spheroids with specific cellular compositions and spatial arrangements, which are essential for replicating the function of native tissues. The integration of various cell types within hydrogel matrices using inkjet printing has led to the development of more physiologically relevant tissue models, advancing the field of organ transplantation and regenerative medicine. Furthermore, the droplet-based printing applied to microarrays can be used for high-throughput spheroid and tissue formation/culture, which can ultimately improve assay throughput and reproducibility for high-throughput, predictive screening of compounds [16, 61, 62].

Vascularization is another critical aspect of tissue engineering, as it ensures the supply of nutrients and oxygen to engineered tissues. Inkjet printing has been instrumental in creating intricate vascular networks within hydrogel constructs [63]. By accurately depositing vascular cells and endothelial growth factors within hydrogels, inkjet printing facilitates the formation of capillary-like structures [64]. This capability is vital for engineering larger tissue constructs, as it addresses the challenge of nutrient diffusion, which is a major limitation in tissue engineering [65]. The development of vascularized tissue constructs through inkjet printing holds great potential for creating fully functional organ replacements.

**Fig. 5:**
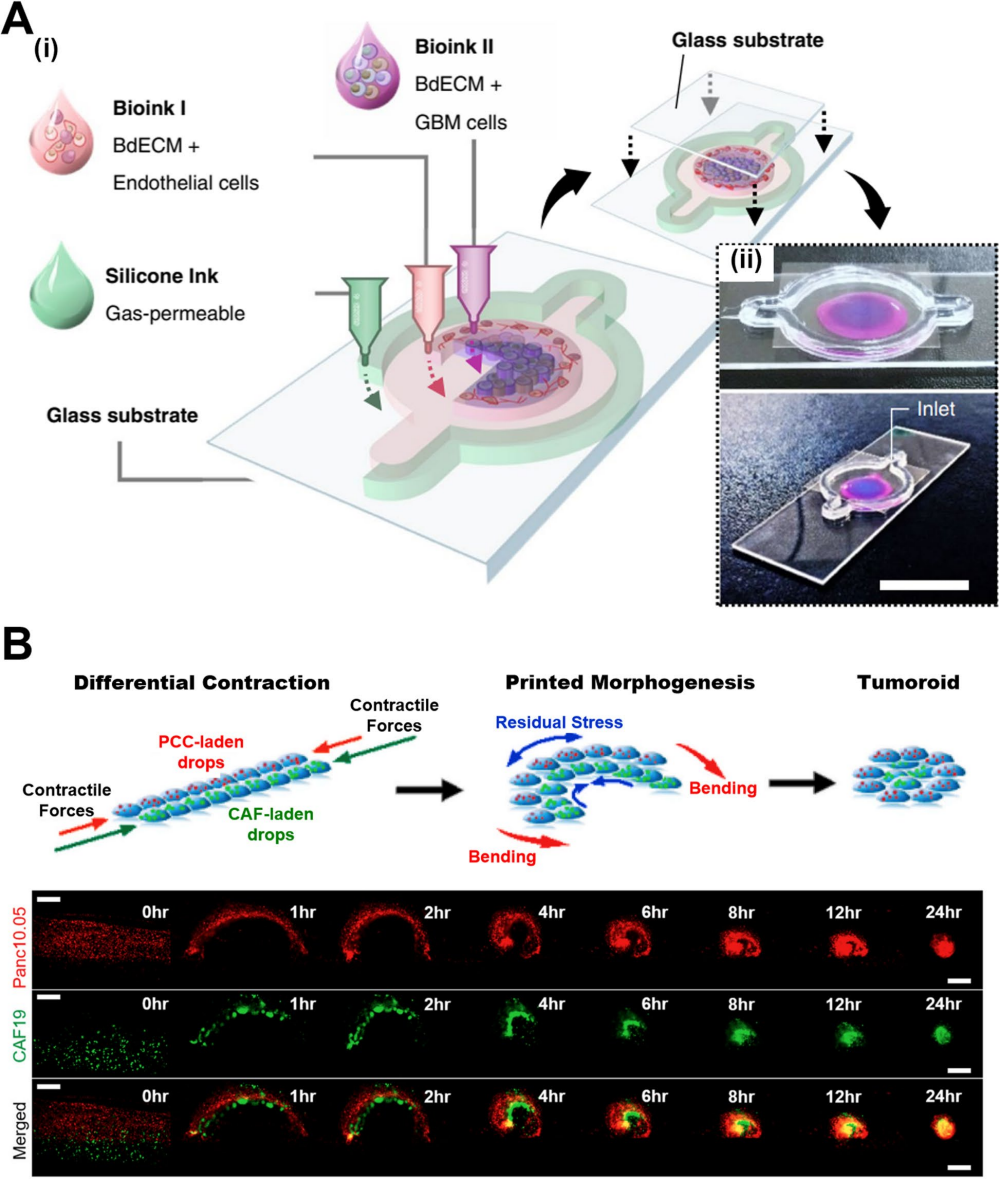
(**A**) (i) Schematic illustration of the bioinks used to fabricate a Glioblastoma (GBM)-on-a-chip model; (ii) Mock representation of bioink compartments of brain dECM laden with HUVECs (depicted in magenta), and brain dECM with GBM cells (blue). Modified from [66] with permission from Nature Portfolio (**B**) Schematic of inkjet printing of cell-laden interpenetrating-polymer inks along with the hypothesized mechanism of tissue compaction and the corresponding 0–24 h time-lapse images showing the active shrinking of printed structure (green: CAF, red: Pacn10.05, scale bar: 500um). Reprinted from [67] under a Creative Commons license

Inkjet printing of hydrogels has also found significant applications in disease modeling, particularly in cancer research and tumor modeling. As shown in Fig. 5A, a study demonstrated a printed human-glioblastoma-on-a-chip that reconstituted the glioblastoma tumor consisting of patient-derived tumor cells, vascular endothelial cells, and decellularized extracellular matrix from brain tissue in a compartmentalized cancer–stroma concentric-ring structure that sustains a radial oxygen gradient while also recapitulating the structural, biochemical and biophysical properties of the native tumors [66]. The technology allows for the fabrication of three-dimensional tumor models that replicate the complex microenvironment of cancerous tissues with cell–cell, cell–matrix, and cell-fluid interactions relevant to the tumor microenvironment (TME) in vivo [63]. By precisely controlling the spatial distribution of cancer cells and surrounding stroma within hydrogels, inkjet printing enables the creation of more accurate and reproducible tumor models compared to traditional two-dimensional cultures [63]. Additionally, a study has developed a new method to rapidly create tumor models with tumor-stroma interface at extremely high cell density, so-called tumoroid, by inkjet printing of multi-line bioinks [67]. The mechanism of the method is that cellular contractile force can significantly remodel the cell-laden polymer matrix to form densely packed tissue-like constructs, as illustrated in Fig. 5B. Moreover, a new concept of digital cell printing is demonstrated to create more complex structures beyond the current 3D printing of hydrogel techniques. This advancement is pivotal for creating a more reliable therapeutic screening platform for preclinical drug evaluation, drug delivery, tumor cell metabolism, signaling pathways, role of integrins and fibronectin.

Apart from cancer modeling, inkjet printing of hydrogels has been used to create multi-layer models of lung cells to aid in the understanding of diseases such as pulmonary fibrosis as well as how lung tissue reacts to inhaled fine particulates. This technique has been demonstrated both in an organ-on-a-chip context as well as in a simple multi-layer contained print using a combination of collagen-based inks as well as inks based on cell culture media [68, 69]. In this work, researchers were able to print up to three layers of varying lung tissue types to create their models, which were found to have significantly increased gene expression as compared to the more traditional 2D cell models. The ability to directly print lung disease models that exhibit similar behavior to real tissue paves the way for an improved understanding of these diseases and how they may be treated. In addition to the study of diseased tissue, further work was conducted to better understand the behavior of lung tissue in the presence of inhaled debris such as dust. These printed lung tissues were found to exhibit similar behavior to natural alveolar tissue when exposed to these materials, further demonstrating their ability to mimic natural lung tissue [70].

As inkjet printing cannot be used with high-viscosity materials owing to limitations on jetting ability, it has limited use in the creation of large-scale 3D structures. However, it has been combined in recent years with more traditional forms of 3D printing, primarily melt electrowriting. This technique combines heat-based extrusion with the presence of an electric field to promote adhesion to a printing surface. This method has been used to print a scaffold structure onto which an inkjet-printed bioink is deposited and used to seed the scaffold structure with cells [71]. This method has seen increasing use, particularly for the creation of bone and cartilage-related models [72–74]. While this has been its predominant use, the technique has also been utilized to create heart models. One study found that it was possible to print mature cardiac cells onto a prepared printed scaffold which, after culturing, contracted in a comparable way and speed to that of normal tissue [75]. This method allows both printing methods to contribute their unique strengths, with the high throughput of extrusion-based printing which lacks precision coupled with the high precision and controllability of inkjet which cannot make significant 3D structures.

Overall, by precisely controlling the deposition of hydrogels laden with therapeutic agents, growth factors, or cells, inkjet printing facilitates the development of customized therapies tailored to individual patient needs. For instance, engineered tissues can be designed to promote wound healing by delivering bioactive compounds that stimulate tissue regeneration or serve as disease models for testing drug responses. Furthermore, the ability to integrate multiple cell types into defined patterns within hydrogels allows for the creation of more physiologically relevant tissue models, enhancing the understanding of disease mechanisms and enabling more effective drug screening platforms. The layer-by-layer precision of inkjet printing enables the creation of tissue constructs with embedded cells and bioactive molecules, offering targeted and localized tissue repair and regeneration. These living constructs can be leveraged to heal chronic wounds, replace damaged cartilage, or even restore organ function, providing a more natural and effective solution than traditional treatments. As such, inkjet printing of hydrogels not only holds promise for advancing regenerative medicine by providing solutions for tissue replacement and repair but may also play a pivotal role in the development of personalized medicine, where treatments are optimized for individual patient profiles, thereby improving therapeutic outcomes and reducing healthcare costs.

The future of inkjet printing in tissue engineering is geared toward enhancing the complexity and functionality of printed constructs. Ongoing research is focused on improving the resolution of printing, developing more advanced hydrogel inks, and integrating multiple cell types to create more sophisticated tissue models [16]. The potential to print entire organs for transplantation remains a long-term goal. Additionally, the integration of sensors and monitoring systems within printed tissues could provide real-time insights into tissue function and response to treatments. As the biomaterial inks become more complex, however, an increased difficulty in printing may arise. Studies have shown that the aggregation of cells and other ink additives can become sufficient to clog the nozzle during long prints resulting in decreased print quality or the inability to print entirely [76]. Although this phenomenon can be mitigated to some degree using inkjet circulation or by ink additives such as polyvinylpyrrolidone, the pursuit of enhanced ink additives and capabilities must be carefully balanced to ensure that printability remains sufficient to allow a high level of architecture control [77].

### Inkjet printing of hydrogel in drug delivery

Drug delivery systems have always been at the nexus of pharmaceutical research, aiming to optimize therapeutic outcomes by ensuring precise dosage, targeted delivery, and controlled release of active pharmaceutical ingredients [78]. In this context, hydrogels, with their unique physicochemical properties, biocompatibility, and ability to encapsulate and release bioactive molecules in a controlled manner, have emerged as a promising material for developing advanced drug delivery platforms [79]. Their biocompatibility, tunable physicochemical properties, and capacity to respond to external stimuli make them particularly suited for delivering a wide range of therapeutics, from small molecules to large biologics.

**Fig. 6:**
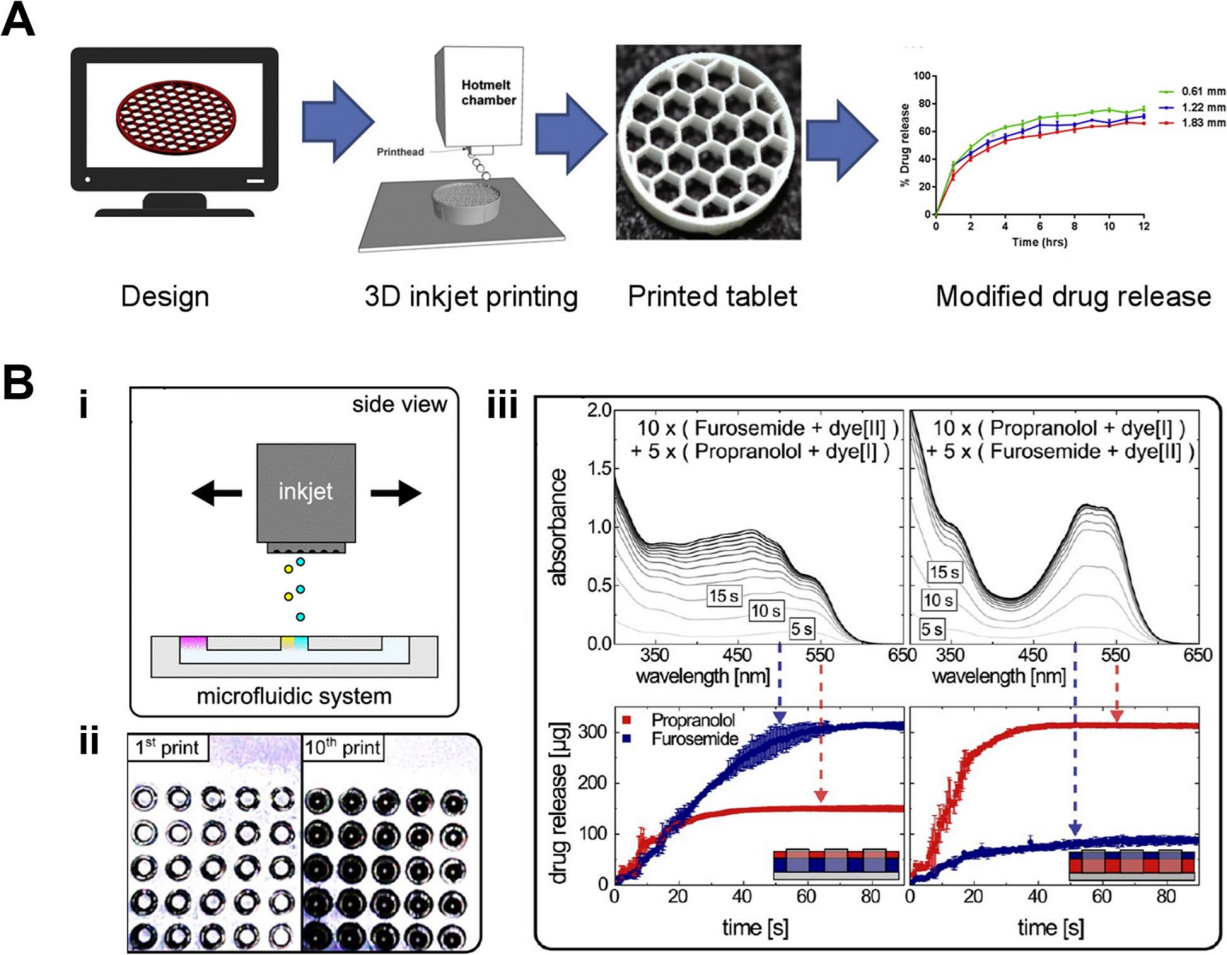
**A** 3D inkjet printing of tablets for controlled and tunable drug release. Reprinted from [83] under a Creative Commons license; **B** Consumer-Grade Inkjet Printer for personalized medicine. Reprinted from [81] under a Creative Commons license—(i) Schematic of the inkjet system during the reactant loading of the microfluidic system; (ii) Optical microscopy images of the micro-containers after the 1st and 10th print, which demonstrates the possibility to do tailored loading with minimal waste; (iii-top) Temporal drug release spectra from microcontainers loaded with first 10 doses/layers of either propranolol or furosemide and then 5 doses/layers of the other drug. (iii-bottom) Using the unmixed signals, the temporal spectra are decomposed to visualize the release kinetics for the individual drugs in the two loading examples

3D printing has emerged as a transformative technology for drug delivery applications due to reasons such as personalized medicine, on-demand production, complex dosage forms, and reduction in costs. Among all the printing methods, inkjet printing, a digital manufacturing technique renowned for its precision, versatility, and scalability, offers the potential to create customized drug delivery devices with unparalleled precision in drug loading and spatial distribution. It could not only customize and tailor drug dosages according to the specific needs of a patient [80, 81] but also allow drug delivery systems with specific release profiles [82, 83], such as delayed or sustained release, as shown in Fig. 6A. This is of importance in scenarios where a gradient of drug concentration (as demonstrated in Fig. 6B) or co-delivery of multiple therapeutics in defined ratios can enhance therapeutic outcomes [81].

Inkjet printing of hydrogels has emerged as a revolutionary approach in the development of controlled and sustained-release drug-delivery systems [79, 82, 83]. This technology enables the precise deposition of therapeutic agents within hydrogel matrices, allowing for the fine-tuning of drug release profiles. The versatility of inkjet printing in manipulating the composition, structure, and geometry of hydrogels results in customizable release kinetics, which can be tailored to meet specific therapeutic needs. For instance, the gradual degradation of hydrogel structures can be engineered to achieve a sustained release of drugs over extended periods, reducing the frequency of drug administration and improving patient compliance [2]. This approach is particularly beneficial for chronic conditions where long-term medication is required.

The precision of inkjet printing in hydrogel fabrication offers significant advantages in targeted drug delivery. By controlling the spatial distribution of drugs within hydrogels, inkjet printing facilitates the localized delivery of therapeutics to specific sites within the body [84], minimizing systemic side effects. This targeted approach is particularly valuable in cancer therapy, where it is crucial to deliver high concentrations of chemotherapeutic agents directly to tumor sites while sparing healthy tissues. Additionally, the incorporation of targeting ligands or antibodies into hydrogel formulations can further enhance the specificity of drug delivery, ensuring that therapeutics are preferentially released at the desired target sites.

Moreover, inkjet-printed hydrogels are designed to release drugs in response to specific physiological stimuli, such as pH changes, temperature fluctuations, or the presence of certain biomolecules [85, 86]. The incorporation of responsive materials into hydrogel matrices enables the creation of smart drug delivery systems that can autonomously regulate drug release based on the body's needs. For example, pH-sensitive hydrogels can be used to release drugs in response to the acidic environment of tumors or inflamed tissues [87]. Magnetic hydrogels showing magneto-responsiveness can facilitate drug release in response to a magnetic field, which has been used in hyperthermia treatment [88]. These responsive systems hold great promise for improving the efficacy and safety of treatments, particularly in the management of diseases with dynamic and complex pathologies.

The future of inkjet-printed hydrogel drug delivery systems lies in further enhancing their precision, responsiveness, and biocompatibility. Advances in ink formulations, printing technologies, and material science are expected to enable the fabrication of more complex and multifunctional hydrogel systems. The integration of biosensors within these systems could provide real-time monitoring of drug release and tissue responses [89], paving the way for personalized and adaptive drug delivery strategies. Additionally, the exploration of novel stimuli-responsive materials and targeting strategies will expand the capabilities of inkjet-printed hydrogels in addressing a wide range of therapeutic challenges.

### Inkjet printing of hydrogels for bioelectronics

**Fig. 7:**
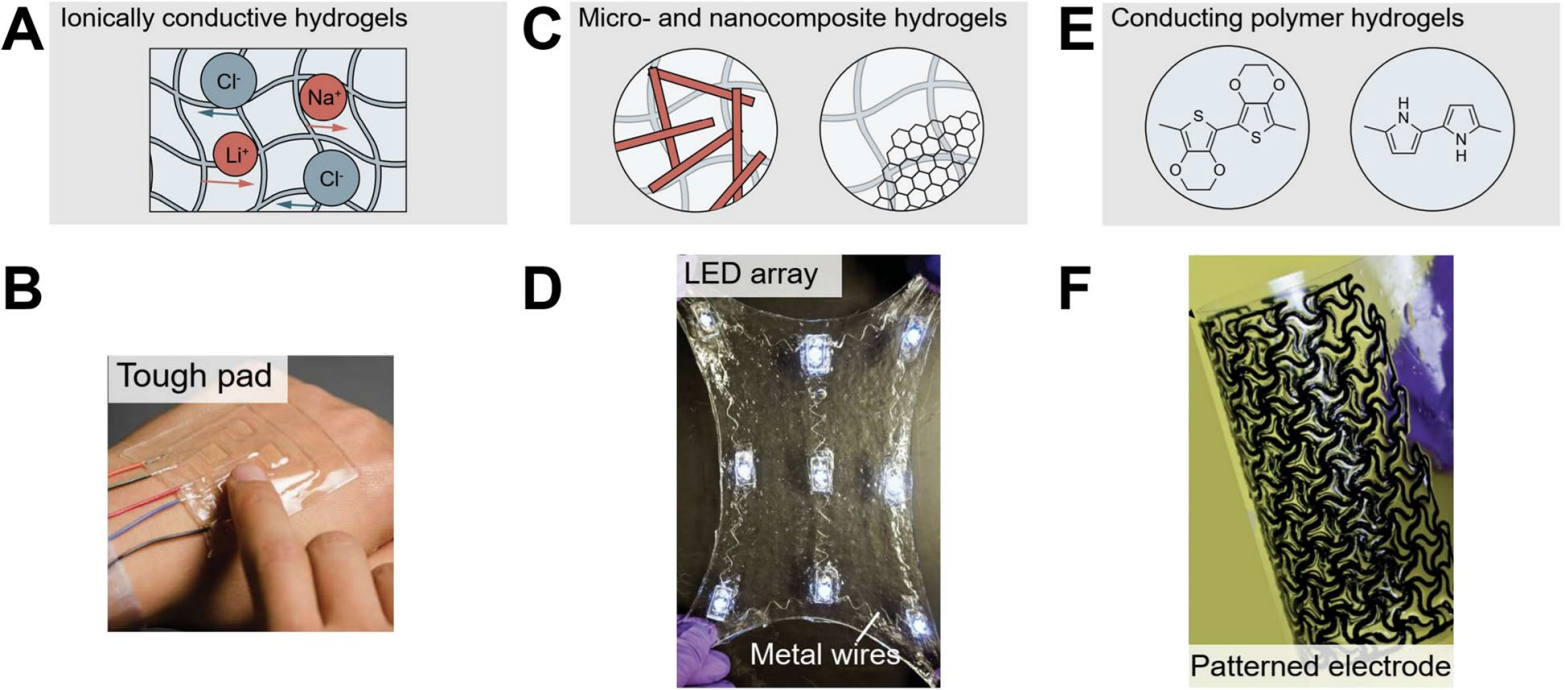
Hydrogel electronics based on (**A**) the addition of ionic salts in the hydrogels to achieve ionically conductive hydrogels and one of its examples (**B**) touch pad. **C** The incorporation of electrically conductive micro or nanocomposites to endow electronic conductivity and one of its examples (**D**) skin patches enabled by incorporating wavy titanium wires in PAAmalginate hydrogel. **E** The introduction of conducting polymers into the polymer networks of hydrogel to enhance electronic conductivity and one of the examples (**F**) patterned PEDOT:PSS hydrogel electrode. Modified from [92] with permission from Elsevier

In the rapidly evolving landscape of bioelectronics, the integration of hydrogels with electronic components has emerged as a groundbreaking approach, bridging the gap between rigid electronics and soft biological tissues [90]. Hydrogels, with their high water content and biocompatibility, mimic the physiological environment, making them ideal candidates for bioelectronic interfaces [91]. The emergence of 3D printing has further revolutionized this domain in fabricating hydrogel-based bioelectronic devices. Due to their superior softness, responsiveness, and biocompatibility, hydrogels are being intensively investigated for versatile functions in stretchable devices and machines, including, actuators, electronics, and water harvesters. A field named hydrogel machines has rapidly evolved [92], exploiting hydrogels as key components for devices and machines, as displayed in Fig. 7. On top of that, the true potential of hydrogel-based bioelectronics has been unlocked with the advent of inkjet printing technology.

Inkjet printing has emerged as a prevalent technique for free-form, high spatial resolution, and multiple materials deposition in printed electronics [93]. When applying hydrogels in the realm of bioelectronics, allows for the deposition of minute droplets to create complex patterns and structures, enabling the fabrication of devices with high spatial resolution. This is particularly crucial for bioelectronic applications where the precise placement of sensors, electrodes, or bioactive compounds can significantly enhance device performance. From wearable devices that monitor physiological parameters to implantable devices that modulate neural activity, the combination of inkjet printing and hydrogels is paving the way for a new generation of bioelectronic devices [94]. These devices are not only more adaptable and user-friendly but also offer the potential for individualized treatments tailored to a patient's specific needs.

Inkjet printing allows for the precise patterning of conductive hydrogels that can be used to create flexible and stretchable electronic circuits, which are integral to the new generation of medical devices. These bioelectronic devices, ranging from biosensors to implantable electrodes, benefit from the inherent biocompatibility and soft mechanics of hydrogels, ensuring minimal invasiveness and enhanced integration with biological tissues. Inkjet-printed hydrogel bioelectronics can be tailored for real-time monitoring of physiological signals with high sensitivity and specificity, offering new avenues for patient monitoring and diagnostics. Furthermore, the versatility of inkjet printing enables the incorporation of various functional materials into hydrogels, allowing for the fabrication of devices that can sense, stimulate, or even treat tissues through electrical signals. Inkjet printing of hydrogels for bioelectronics represents a transformative approach in the medical device industry, promising to enhance the efficacy and comfort of future healthcare solutions.

Additionally, hydrogel sensors and actuators have been extensively reviewed [95]. The working principle of hydrogel-based soft actuators is that the hydrogel has the swell/shrink capability depending on the water content, which allows various actuations and motions. This water content is responsive and sensitive to multiple external stimuli, such as heat, electricity, magnetism, strain, pH, and chemical reactions, making them ideal for wearable or implantable sensors. A study demonstrated the use of inkjet printing to create a hydrogel-based glucose sensor [96]. The sensor was able to detect glucose levels in real-time and was biocompatible for implantation. Another study highlighted the creation of a neural interface using hydrogel that could both record neural signals and stimulate neurons [97]. Similarly, their optimized swelling/shrinkage properties can be used as sensors to convert external stimuli into electromechanical signals [98]. In addition, more and more studies have attempted to enhance the electrical conductivity of hydrogels, which have been used to achieve various hydrogel electronics [99]. The field of hydrogel machines will ultimately replace or complement or replace many conventional devices based on rigid materials by integrating hydrogels that can potentially minimize physical and physiological mismatches with biological organs and tissues.

Soft robotics are made with soft and compliant materials and are often inspired by biological structures to achieve desired flexibility and adaptability to dynamic environments [100]. Hydrogel materials allow the mobility of soft robots in dynamic environments because they can passively deform and adapt to the surrounding shape. Additionally, soft robots possess high impact resistance because they distribute stress over a large area. Advances in hydrogels and inkjet printing technologies have enabled the design of soft robots with sophisticated capabilities, such as grasping a fragile object [101] or moving on various shapes of substrates [102].

Soft robots serve as a promising approach in the biomedical area [103]. For example, in minimally invasive surgery and endoscopy, robots operate inside a live body and physically contact a patient. Recently, Hu et al. have developed a soft small-scale robot that can roll into an ex-vivo chicken tissue [104]. Moreover, soft robotics can be used in human body rehabilitation (e.g., knee, foot, hand, etc.) and assistance. A powerful example is provided by the I-support arm designed to help older people with shower tasks [105], such as bathing.

In the last few years, a growing interest in additive manufacturing (AM) technologies has been broadly utilized in soft robotics. Because of several intrinsic attributes of AM, such as the possibility to fabricate objects with very complex geometries and the ability to use multiple materials in a single printing process. Besides, the expansion of the materials also accelerates the growing need for AM of soft materials.

In direct inkjet printing, inkjet printheads jet the liquid resins onto the substrate before solidifying. One of the advantages of inkjet printing is the simultaneous deposition of multi-materials. Therefore, a printed part can have selective mechanical properties, opacities, and colors [106]. For example, a robot can be constructed with inkjet materials with different mechanical stiffness (i.e., elastic modulus), enabling a programmable mechanical response [107] to temperature, electrical stimuli, or oscillating magnetic fields. Umedachi et al. have designed a soft worm with variable friction feet [108], enabling worm-like locomotion through repeated tendon actuation. However, other soft robotic applications require even softer and more stretchable materials than commercially available inkjet materials. In addition, the physics of droplet deposition and formation needs more in-depth studies, including processing conditions, ink formulation, and water-matrix interaction, which ultimately affect the qualities of printed products.

Compared to traditional fabrication methods, inkjet printing offers more freedom to design complex geometries and enables the production of components that combine multiple materials. Using this principle makes it possible to create mechanical devices combining soft deformable parts with more rigid elements, suitable for fabricating hybrid soft-rigid robotic systems, benefiting both from soft-compliant elements and structural reinforcements.

## Conclusion and discussion

Inkjet printing of hydrogels has emerged as a pivotal technology in the realm of biomedical engineering, offering unparalleled precision and versatility in fabricating complex tissue constructs. This review has explored the multifaceted applications of this technology, particularly in the development of tissue engineering, vascularization strategies, disease models, drug delivery systems, and bioelectronics. The critical aspects of ink selection, jetting behavior, and the optimization of printing parameters are also reviewed. This includes considerations like viscosity, surface tension, and curing mechanisms, which are essential for achieving desired structural and functional properties in the printed constructs.

The ability of inkjet printing to precisely deposit cells and biomaterials has been instrumental in tissue engineering, including creating organoids, spheroids, vascular networks, and tumor models [18, 109]. These 3D structures mimic the microenvironment of human tissues, providing a more accurate platform for studying disease progression and drug response. The application of inkjet printing in creating disease models, particularly for cancer research, has opened new avenues for understanding tumor biology and testing therapeutics. The technology allows for the fabrication of tumor models with controlled microenvironments, enabling the study of cancer cell behavior and drug efficacy in a more physiologically relevant context.

Inkjet printing has also been utilized in developing novel drug delivery systems, including controlled and sustained release systems, targeted drug delivery, and responsive drug delivery systems [110]. The precision of inkjet printing allows for the fabrication of delivery systems with specific geometries and compositions, enabling more effective and targeted therapeutic interventions.

Compared to traditional tissue engineering methods, 3D printing offers unique benefits, including the ability to control the microenvironment of cells with high resolution. This control is essential for directing cell behavior, differentiation, and maturation into functional tissues. Unlike scaffold-based or self-assembly approaches, 3D printing can create predefined structures with vascular networks, enabling the engineering of thicker, more complex tissues with their nutrient supply systems. To maintain these advantages, the field must focus on developing inks that support cell growth and differentiation while remaining printable. Additionally, integrating sensors within printed constructs to monitor tissue development and health in real-time could further enhance the capabilities of printed tissues.

## Challenges and opportunities

Despite the potential advantage, designing the DOD printing process for hydrogel materials remains heuristic due to the intricate properties of hydrogels and the complex requirements of 3D printing. Challenges primarily arise from controlling viscosity and rheology. Hydrogels often exhibit complex rheological behaviors, requiring precise adjustments to ensure smooth ejection through the nozzles while maintaining the structural integrity of the printed material. Another significant challenge is maintaining the viability and functionality of cells when printing hydrogels that contain living cells. Additionally, the mechanical properties of hydrogels, such as strength and elasticity, also pose challenges as they must be tailored to match the specific requirements of the intended application, a task complicated by the inherently soft and porous nature of hydrogels.

The process of curing or crosslinking the hydrogel after printing requires careful control. However, there is a limited mechanistic understanding of the behavior of polymer drops after deposition, as most DOD printing studies have focused on finding reliable jetting conditions for a given polymer ink [111, 112]. The complex fluid-matrix interaction during curing, which determines the functional properties of the printed hydrogel products, is poorly understood. Although the evaporation of sessile drops and colloidal drops (i.e., liquid drops where insoluble particles are suspended) has been studied extensively [113], the evaporation of liquid or colloidal drops is not directly relevant to the dehydration of polymer drops due to the porous polymer matrix and subsequent fluid-matrix interactions. Furthermore, the interaction becomes more complicated when particulates, such as metal particles, biological cells, and drug compounds, are added to the hydrogel, limiting DOD printing of hydrogels to trial-and-error approaches.

Previous studies have investigated single-drop and multi-drop dehydration that considers water-matrix and drop-drop interactions during the curing process [49, 50]. An extensive study proposed a scaling law for the prediction of the particle transport within the hydrogel during curing to enable the controllable particle distribution within printed hydrogels [51]. However, this study purely assumes that particle transport resembles interstitial water flow. Studies under practical conditions are still needed to give us a better understanding of particle transport within the hydrogel. For example, pore size and distribution of the polymer matrix play a crucial role in determining how particles move. Polymer-particle interactions, including hydrophobicity and hydrophilicity, can also influence particle transport. Swelling and shrinking of the porous polymer matrix also changes the pore structure dynamically. Therefore, particle transport within hydrogels is a research gap that needs to be addressed. It is possible that a machine learning model could be trained on simulations of printed hydrogels and then used to better predict and design future DOD printing experiments. The use of machine learning in inkjet printing is not a novel idea and has already been demonstrated with some success in work aiming to predict the cell content of a given printed droplet [114]. Though additional work is needed to fully determine the feasibility of this idea, it has the potential to yield significant improvements in the inkjet printing of hydrogels.

Moreover, integrating 3D printing of hydrogel with another rapidly advancing technology, artificial intelligence (AI), offers opportunities to revolutionize the field of tissue engineering and regenerative medicine [115]. AI can optimize 3D printing parameters for individual patients, predicting the best tissue constructs for repair or disease modeling. AI may also be able to predict the behavior of various materials under different conditions, aiding in the selection of the most suitable biomaterials and bioink formulations for specific applications. AI's flexibility allows it to be integrated with other technologies, such as microfluidics for creating complex tissue models with precise control over the microenvironment, or augmented reality for visualizing the 3D printing process in real-time. This integration not only promises to advance the field of regenerative medicine but also opens new possibilities for drug testing, disease modeling, and personalized medicine.

Overall, inkjet printing of particulate-laden hydrogels holds significant promise in advancing biomedical applications [116]. Its ability to fabricate complex, multi-cellular structures with high precision makes it a valuable tool in tissue engineering, disease modeling, and drug delivery. Challenges include the need for precise control of viscosity and rheology to ensure smooth ejection and structural integrity, and the maintenance of cell viability during and after printing. Achieving high resolution and accuracy in printed structures, while tailoring the mechanical properties of hydrogels to match application-specific requirements [117]. Moreover, the curing and crosslinking process must be carefully managed to balance structural stability with cell health and achieve the desired particulate pattern post-printing and curing. Addressing these challenges requires a multidisciplinary approach, combining insights and advancements from material science, biology, engineering, and technology. The design, synthesis, and testing of inkjet printing for bioapplications is not a simple task. Coupling the complexity of jetting liquids with the necessary considerations to print cultured cell solutions presents an involved process that cannot be taken for granted. These difficulties may restrict future work in inkjet printing of hydrogel solutions to some degree owing to issues with ink formulation or physical limits on viscosity or other material properties. Despite these challenges, as the technology continues to evolve, inkjet printing of particulate-laden hydrogels is expected to play an increasingly vital role in a wide range of biomedical applications, including personalized medicine and advanced therapeutic strategies.

## Data Availability

Not applicable.

## References

[1] Lee JH, Kim HW (2018). Emerging properties of hydrogels in tissue engineering. J Tissue Engine.

[2] Li J, Mooney DJ (2016). Designing hydrogels for controlled drug delivery. Nat Rev Mat.

[3] Keplinger C, Sun J-Y, Foo CC, Rothemund P, Whitesides GM, Suo Z (2013). Stretchable, transparent, ionic conductors. Science.

[4] Fawaz J, Wang K, Almansoori A. Energy gas storage in porous polymers. In: Mittal V, editor. Polymers for energy storage and conversion. Wiley, Inc.; 2013. p. 215–48. 10.1002/9781118734162.

[5] Cheng Y (2012). Electroaddressing functionalized polysaccharides as model biofilms for interrogating cell signaling. Adv Func Mater.

[6] Liu Z, Takeuchi M, Nakajima M, Hasegawa Y, Huang Q, Fukuda T (2016). Shape-controlled high cell-density microcapsules by electrodeposition. Acta Biomaterialia.

[7] Liu C, Xu N, Zong Q, Yu J, Zhang P (2021). Hydrogel prepared by 3D printing technology and its applications in the medical field. Colloid Interface Sci Commun.

[8] Zhang J (2019). Silk particles, microfibres and nanofibres: a comparative study of their functions in 3D printing hydrogel scaffolds. Mat Sci Engine C.

[9] Zhao D (2021). 3D printing method for tough multifunctional particle-based double-network hydrogels. ACS Appl Mat Interfaces.

[10] Baumann B (2017). Kontrolle der Freisetzungskinetik von Nanopartikeln aus 3D-gedruckten Hydrogelgerüsten. Angewandte Chemie.

[11] Miar S, Shafiee A, Guda T, Narayan R, Ovsianikov A, Yoo J, Mironov V (2018). Additive Manufacturing for Tissue Engineering. 3D Printing and Biofabrication.

[12] Gardan J. Virtual and Physical Prototyping Smart materials in additive manufacturing: state of the art and trends. 2018. 10.1080/17452759.2018.1518016.

[13] Yeong WY, Chua CK. Bioprinting: principles and applications. World Scientific Publishing Co Inc.; 2014. 10.1142/9193.

[14] Dou C, Perez V, Qu J, Tsin A, Xu B, Li J (2021). A state-of-the-art review of laser-assisted bioprinting and its future research trends. ChemBioEng Rev.

[15] Ablanedo Morales P (2023). Thermal inkjet bioprinting drastically alters cell phenotype. Biofabrication.

[16] Li X, et al. Inkjet bioprinting of biomaterials. In: Chemical Reviews. Vol. 120. American Chemical Society; 2020. p. 10793–833. 10.1021/acs.chemrev.0c00008. 10.1021/acs.chemrev.0c00008 32902959

[17] Advincula RC, Ryan Dizon JC, Caldona EB, Andrew Viers R, Dave FC, Siacor FC, Espera AH (2021). On the progress of 3D-printed hydrogels for tissue engineering. MRS Commun.

[18] Negro A, Cherbuin T, Lutolf MP (2018). 3D Inkjet printing of complex, cell-laden hydrogel structures. Sci Rep.

[19] Park JA (2017). Freeform micropatterning of living cells into cell culture medium using direct inkjet printing. Scient Rep.

[20] Li L (2015). A nanostructured conductive hydrogels-based biosensor platform for human metabolite detection. Nano Lett.

[21] Huang H, Yu Y, Hu Y, He X, BerkUsta O, Yarmush ML (2017). Generation and manipulation of hydrogel microcapsules by droplet-based microfluidics for mammalian cell culture. Lab on a Chip.

[22] Mishra B, Upadhyay M, Reddy Adena SK, Vascant BG, Muthu MS (2017). Hydrogels: an introduction to a controlled drug delivery device, synthesis and application in drug delivery and tissue engineering. Austin J Biomed Eng.

[23] Suntornnond R, Ng WL, Huang X, Yeow CHE, Yeong WY (2022). Improving printability of hydrogel-based bio-inks for thermal inkjet bioprinting applications via saponification and heat treatment processes. J Mat Chem B.

[24] Malda J (2013). 25th anniversary article: engineering hydrogels for biofabrication. In Adv Mat.

[25] Chimene D, Kaunas R, Gaharwar AK. Hydrogel bioink reinforcement for additive manufacturing: a focused review of emerging strategies. In: Advanced Materials. Vol. 32. Wiley-VCH Verlag; 2020. 10.1002/adma.201902026. 10.1002/adma.201902026 31599073

[26] Jungst T, Smolan W, Schacht K, Scheibel T, Groll J. Strategies and molecular design criteria for 3D printable hydrogels. In: Chemical Reviews. Vol. 116. American Chemical Society; 2016. p. 1496–539. 10.1021/acs.chemrev.5b00303. 10.1021/acs.chemrev.5b00303 26492834

[27] Hölzl K, Lin S, Tytgat L, Van Vlierberghe S, Gu L, Ovsianikov A. Bioink properties before, during and after 3D bioprinting. In: Biofabrication. Vol. 8. Institute of Physics Publishing; 2016. 10.1088/1758-5090/8/3/032002. 10.1088/1758-5090/8/3/032002 27658612

[28] Chen Y (2020). 3D Bioprinting of shear-thinning hybrid bioinks with excellent bioactivity derived from gellan/alginate and thixotropic magnesium phosphate-based gels. J Mat Chem B.

[29] Guimarães CF, Gasperini L, Marques AP, Reis RL. The stiffness of living tissues and its implications for tissue engineering. In: Nature Reviews Materials. Vol. 5. Nature Research; 2020. p. 351–70. 10.1038/s41578-019-0169-1.

[30] Patel NR, Whitehead AK, Newman JJ, Caldorera-Moore ME (2017). Poly(ethylene glycol) hydrogels with tailorable surface and mechanical properties for tissue engineering applications. ACS Biomater Sci Eng.

[31] Unagolla JM, Jayasuriya AC. Hydrogel-based 3D bioprinting: A comprehensive review on cell-laden hydrogels, bioink formulations, and future perspectives. In: ApplieD Materials Today. vol. 18. Elsevier Ltd; 2020. 10.1016/j.apmt.2019.100479. 10.1016/j.apmt.2019.100479 PMC7414424 32775607

[32] Zhu J, Marchant RE (2011). Design properties of hydrogel tissue-engineering scaffolds. Exp Rev Med Dev.

[33] Chaudhary S, Chakraborty E. Hydrogel based tissue engineering and its future applications in personalized disease modeling and regenerative therapy. In: Beni-Suef University Journal of Basic and Applied Sciences. Vol. 11. Springer Science and Business Media Deutschland GmbH; 2022. 10.1186/s43088-021-00172-1. 10.1186/s43088-021-00172-1 PMC8725962 35005036

[34] Xu J, et al. Advances in the research of bioinks based on natural collagen, polysaccharide and their derivatives for skin 3D bioprinting. In: Polymers. Vol. 12. MDPI AG; 2020. 10.3390/polym12061237. 10.3390/polym12061237 PMC7362214 32485901

[35] Shah MA, Lee DG, Lee BY, Hur S. Classifications and applications of inkjet printing technology: a review. In: IEEE Access. Vol. 9. Institute of Electrical and Electronics Engineers Inc., 2021; p. 140079–102. 10.1109/ACCESS.2021.3119219.

[36] Xu C, Zhang M, Huang Y, Ogale A, Fu J, Markwald RR (2014). Study of droplet formation process during drop-on-demand inkjetting of living cell-laden bioink. Langmuir.

[37] Guo Y, Patanwala HS, Bognet B, Ma AWK (2017). Inkjet and inkjet-based 3D printing: connecting fluid properties and printing performance. Rapid Prototyping J.

[38] Furbank RJ, Morris JF (2004). An experimental study of particle effects on drop formation. Phys Fluids.

[39] Bognet B, Guo Y, Ma AWK (2016). Controlling system components with a sound card: a versatile inkjet fluid testing platform. Rev Scient Instruments.

[40] Shi J, Wu B, Li S, Song J, Song B, Lu WF (2018). Shear stress analysis and its effects on cell viability and cell proliferation in drop-on-demand bioprinting. Biomed Phys Engine Expr.

[41] Li H, Tan C, Li L (2018). Review of 3D printable hydrogels and constructs. Mat Design.

[42] Teo MY, Kee S, RaviChandran N, Stuart L, Aw KC, Stringer J (2020). Enabling free-standing 3D hydrogel microstructures with microreactive inkjet printing. ACS Appl Mat Interfaces.

[43] He Y, Wang F, Wang X, Zhang J, Wang D, Huang X (2021). A photocurable hybrid chitosan/acrylamide bioink for DLP based 3D bioprinting. Mat Design.

[44] Janmaleki M, Liu J, Kamkar M, Azarmanesh M, Sundararaj U, Nezhad AS (2021). Role of temperature on bio-printability of gelatin methacryloyl bioink in two-step cross-linking strategy for tissue engineering applications. Biomed Mat.

[45] Gopinathan J, Noh I. Click chemistry-based injectable hydrogels and bioprinting inks for tissue engineering applications. In: Tissue Engineering and Regenerative Medicine. Vol. 15. Korean Tissue Engineering and Regenerative Medicine Society; 2018. p. 531–46. 10.1007/s13770-018-0152-8. 10.1007/s13770-018-0152-8 PMC6171698 30603577

[46] Cui X, Dean D, Ruggeri ZM, Boland T (2010). Cell damage evaluation of thermal inkjet printed Chinese hamster ovary cells. Biotechnol Bioengine.

[47] Ouyang L, Yao R, Zhao Y, Sun W (2016). Effect of bioink properties on printability and cell viability for 3D bioplotting of embryonic stem cells. Biofabrication.

[48] Rodriguez MJ, Brown J, Giordano J, Lin SJ, Omenetto FG, Kaplan DL (2017). Silk based bioinks for soft tissue reconstruction using 3-dimensional (3D) printing with in vitro and in vivo assessments. Biomaterials.

[49] Han B (2016). Dropwise gelation-dehydration kinetics during drop-on-demand printing of hydrogel-based materials. Int J Heat Mass Transf.

[50] Cheng C (2020). Water-matrix interaction at the drop-drop interface during drop-on-demand printing of hydrogels. Int J Heat Mass Transfer.

[51] Cheng C, Moon YJ, Hwang JY, Chiu GTC, Han B (2022). A scaling law of particle transport in inkjet-printed particle-laden polymeric drops. Int J Heat Mass Transf.

[52] Zhou K, Sun Y, Yang J, Mao H, Gu Z (2022). Hydrogels for 3D embedded bioprinting: a focused review on bioinks and support baths. J Mat Chem B.

[53] Saroia J, Yanen W, Wei Q, Zhang K, Lu T, Zhang B (2018). A review on biocompatibility nature of hydrogels with 3D printing techniques, tissue engineering application and its future prospective. Bio-Design Manufact.

[54] Li S, Xu LX (2023). X for medicine: exploration and innovation in biomedical engineering. Med-X.

[55] Gyles DA, Castro LD, Silva JOC, Ribeiro-Costa RM (2017). A review of the designs and prominent biomedical advances of natural and synthetic hydrogel formulations. Eur Polymer J.

[56] Brochu ABW, Craig SL, Reichert WM (2011). Self-healing biomaterials. J Biomed Mat Res Part A.

[57] Cui X, Boland T, D'Lima DD, Lotz MK (2012). Thermal inkjet printing in tissue engineering and regenerative medicine. Recent Patents Drug Deliver Formula.

[58] Christensen K, Xu C, Chai W, Zhang Z, Fu J, Huang Y (2015). Freeform inkjet printing of cellular structures with bifurcations. Biotechnol Bioengine.

[59] Ravanbakhsh H, Karamzadeh V, Bao G, Mongeau L, Juncker D, Zhang YS (2021). Emerging technologies in multi-material bioprinting. Adv Mat.

[60] Zips S (2023). Aerosol jet-printed high-aspect ratio micro-needle electrode arrays applied for human cerebral organoids and 3D neurospheroid networks. ACS Appl Mat Interfaces.

[61] Shrestha S, Lekkala VK, Acharya P, Siddhpura D, Lee MY (2021). Recent advances in microarray 3D bioprinting for high-throughput spheroid and tissue culture and analysis. Essays Biochem.

[62] Scoutaris N, Ross S, Douroumis D. Current trends on medical and pharmaceutical applications of inkjet printing technology. In: Pharmaceutical Research. Vol. 33. Springer New York LLC; 2016. p. 1799–816. 10.1007/s11095-016-1931-3. 10.1007/s11095-016-1931-3 27174300

[63] Datta P, Dey M, Ataie Z, Unutmaz D, Ozbolat IT. 3D bioprinting for reconstituting the cancer microenvironment. In: NPJ Precision Oncology. Vol. 4. Nature Research; 2020. 10.1038/s41698-020-0121-2. 10.1038/s41698-020-0121-2 PMC7385083 32793806

[64] Lee VK, Lanzi AM, Ngo H, Yoo SS, Vincent PA, Dai G (2014). Generation of multi-scale vascular network system within 3D hydrogel using 3D bio-printing technology. Cell Mol Bioeng.

[65] Trimm E, Red-Horse K. Vascular endothelial cell development and diversity. In: Nature Reviews Cardiology. Vol. 20. Nature Research; 2023. p. 197–210. 10.1038/s41569-022-00770-1. 10.1038/s41569-022-00770-1 PMC9533272 36198871

[66] Yi HG (2019). A bioprinted human-glioblastoma-on-a-chip for the identification of patient-specific responses to chemoradiotherapy. Nat Biomed Engine.

[67] Cheng C (2023). Inkjet-printed morphogenesis of tumor-stroma interface using bi-cellular bioinks of collagen-poly(N-isopropyl acrylamide-co-methyl methacrylate) mixture. Mat Today Adv.

[68] Kim W, Lee Y, Kang D, Kwak T, Lee H-R, Jung S (2023). 3D inkjet-bioprinted lung-on-a-chip. ACS Biomat Sci Engine.

[69] Kang D, Lee Y, Kim W, Lee HR, Jung S (2023). 3D pulmonary fibrosis model for anti-fibrotic drug discovery by inkjet-bioprinting. Biomed Mat (Bristol).

[70] Kang D, Lee H, Jung S (2022). Use of a 3D inkjet-printed model to access dust particle toxicology in the human alveolar barrier. Biotechnol Bioengine.

[71] Dufour A (2022). Integrating melt electrowriting and inkjet bioprinting for engineering structurally organized articular cartilage. Biomaterials.

[72] Barceló X, Eichholz KF, Gonçalves IF, Garcia O, Kelly DJ (2023). Bioprinting of structurally organized meniscal tissue within anisotropic melt electrowritten scaffolds. Acta Biomater.

[73] Sufaru IG, et al. 3D printed and bioprinted membranes and scaffolds for the periodontal tissue regeneration: a narrative review. In: Membranes. Vol. 12. MDPI; 2022. 10.3390/membranes12090902. 10.3390/membranes12090902 PMC9505571 36135920

[74] Tharakan S, Khondkar S, Ilyas A. Bioprinting of stem cells in multimaterial scaffolds and their applications in bone tissue engineering, In: Sensors. Vol. 21. MDPI; 2021. 10.3390/s21227477. 10.3390/s21227477 PMC8618842 34833553

[75] Iwanaga S (2022). Design and fabrication of mature engineered pre-cardiac tissue utilizing 3D bioprinting technology and enzymatically crosslinking hydrogel. Materials.

[76] Xu H, Salazar DMM, Shahriar M, Xu C (2022). Investigation and characterization of cell aggregation during and after inkjet-based bioprinting of cell-laden bioink. J Manufact Sci Engine.

[77] Ng WL, Huang X, Shkolnikov V, Suntornnond R, Yeong WY (2023). Polyvinylpyrrolidone-based bioink: influence of bioink properties on printing performance and cell proliferation during inkjet-based bioprinting. Bio-Design Manufact.

[78] Sung YK, Kim SW. Recent advances in polymeric drug delivery systems. In: Biomaterials Research. Vol. 24. BioMed Central Ltd; 2020. 10.1186/s40824-020-00190-7. 10.1186/s40824-020-00190-7 PMC7285724 32537239

[79] Dreiss CA (2020). Hydrogel design strategies for drug delivery. Curr Opin Colloid Interf Sci.

[80] Jain V, Haider N, Jain K (2018). 3D printing in personalized drug delivery. Curr Pharm Des.

[81] Mandsberg NK, Højgaard J, Joshi SS, Nielsen LH, Boisen A, Hwu ET (2021). Consumer-grade inkjet printer for versatile and precise chemical deposition. ACS Omega.

[82] Chung JHY, Naficy S, Wallace GG, Naficy S, O'Leary S (2016). Inkjet-printed alginate microspheres as additional drug carriers for injectable hydrogels. Adv Polymer Technol.

[83] Kyobula M (2017). 3D inkjet printing of tablets exploiting bespoke complex geometries for controlled and tuneable drug release. J Contr Rel.

[84] Chou W-H, Gamboa A, Morales JO (2021). Inkjet printing of small molecules, biologics, and nanoparticles. Int J Pharm.

[85] Thirupathi K (2023). Thermosensitive polymer-modified mesoporous silica for pH and temperature-responsive drug delivery. Pharmaceutics.

[86] Vaupel S, Mau R, Kara S, Seitz H, Kragl U, Meyer J (2023). 3D printed and stimulus responsive drug delivery systems based on synthetic polyelectrolyte hydrogels manufactured via digital light processing. J Mat Chem B.

[87] Bami MS, RaeisiEstabragh MA, Khazaeli P, Ohadi M, Dehghannoudeh G (2022). pH-responsive drug delivery systems as intelligent carriers for targeted drug therapy: brief history, properties, synthesis, mechanism and application. J Drug Delivery Sci Technol.

[88] Ganguly S, Margel S. Design of magnetic hydrogels for hyperthermia and drug delivery. In: Polymers. Vol. 13. MDPI; 2021. 10.3390/polym13234259. 10.3390/polym13234259 PMC8659876 34883761

[89] Cao Y, Dumani DS, Hallam KA, Emelianov SY, Ran H (2023). Real-time monitoring of NIR-triggered drug release from phase-changeable nanodroplets by photoacoustic/ultrasound imaging. Photoacoustics.

[90] Calvert P (2009). Hydrogels for soft machines. Adv Mater.

[91] Yuk H, Lu B, Zhao X (2019). Hydrogel bioelectronics. Chem Soc Rev.

[92] Liu X, Liu J, Lin S, Zhao X (2020). Hydrogel machines. Mater Today.

[93] Teo MY (2019). Direct patterning of highly conductive PEDOT:PSS/Ionic liquid hydrogel via microreactive inkjet printing. ACS Appl Mat Interf.

[94] Gao M, Li L, Song Y. Inkjet printing wearable electronic devices. In: Journal of Materials Chemistry C. Vol. 5. Royal Society of Chemistry; 2017. p. 2971–93. 10.1039/C7TC00038C.

[95] Banerjee H, Suhail M, Ren H (2018). biomimetics hydrogel actuators and sensors for biomedical soft robots: brief overview with impending challenges. Biomimetics.

[96] Song J, et al. Hydrogel-based flexible materials for diabetes diagnosis, treatment, and management. In: NPJ Flexible Electronics. Vol. 5. Nature Research; 2021. 10.1038/s41528-021-00122-y.

[97] Almasri RM, Alchamaa W, Tehrani-Bagha AR, Khraiche ML (2020). Highly flexible single-unit resolution all printed neural interface on a bioresorbable backbone. ACS Appl Bio Mat.

[98] Zhou X (2023). Gel-based strain/pressure sensors for underwater sensing: Sensing mechanisms, design strategies and applications. Compos B Eng.

[99] Zhou Q (2021). Mechanically strong and multifunctional hybrid hydrogels with ultrahigh electrical conductivity. Adv Funct Mat.

[100] SachyaniKeneth E, Kamyshny A, Totaro M, Beccai L, Magdassi S (2021). 3D printing materials for soft robotics. Adv Mat.

[101] Shintake J, Rosset S, Schubert B, Floreano D, Shea H (2016). Versatile soft grippers with intrinsic electroadhesion based on multifunctional polymer actuators. Adv Mater.

[102] Hu Y (2017). Electrically and sunlight-driven actuator with versatile biomimetic motions based on rolled carbon nanotube bilayer composite. Adv Funct Mat.

[103] Cianchetti M, Laschi C, Menciassi A, Dario P (2018). Biomedical applications of soft robotics. Nat Rev Mater.

[104] Hu W, Lum GZ, Mastrangeli M, Sitti M (2018). Small-scale soft-bodied robot with multimodal locomotion. Nature.

[105] Manti M, Pratesi A, Falotico E, Cianchetti M, Laschi C (2016). Soft assistive robot for personal care of elderly people. Proc IEEE RAS EMBS Int Confer Biomed Robot Biomechatr.

[106] Vaezi M, Chianrabutra S, Mellor B, Yang S (2013). Multiple material additive manufacturing - Part 1: A review: This review paper covers a decade of research on multiple material additive manufacturing technologies which can produce complex geometry parts with different materials. Virt Phys Prototyp.

[107] Ge Q, Sakhaei AH, Lee H, Dunn CK, Fang NX, Dunn ML. Multimaterial 4D Printing with Tailorable Shape Memory Polymers OPEN. Nature Publishing Group; 2016. 10.1038/srep31110. 10.1038/srep31110 PMC4976324 27499417

[108] Umedachi T, Vikas V, Trimmer BA (2016). Softworms : the design and control of non-pneumatic, 3D-printed, deformable robots. Bioinspir Biomim.

[109] Boland T, Xu T, Damon B, Cui X (2006). Application of inkjet printing to tissue engineering. Biotechnol J.

[110] Boehm RD, Miller PR, Daniels J, Stafslien S, Narayan RJ (2014). Inkjet printing for pharmaceutical applications. Mater Today.

[111] He Y, Yang F, Zhao H, Gao Q, Xia B, Fu J (2016). Research on the printability of hydrogels in 3D bioprinting. Sci Rep.

[112] Nishiyama Y (2009). Development of a three-dimensional bioprinter: construction of cell supporting structures using hydrogel and state-of-the-art inkjet technology. J Biomech Eng.

[113] Bouchenna C, AitSaada M, Chikh S, Tadrist L (2017). Generalized formulation for evaporation rate and flow pattern prediction inside an evaporating pinned sessile drop. Int J Heat Mass Transfer.

[114] Huang X, Ng WL, Yeong WY. Predicting the number of printed cells during inkjet-based bioprinting process based on droplet velocity profile using machine learning approaches. J Intell Manuf. 2023. 10.1007/s10845-023-02167-4.

[115] Zhu Z, Ng DW, Park HS, McAlpine MC (2021). 3D-printed multifunctional materials enabled by artificial-intelligence-assisted fabrication technologies. Nat Rev Mat.

[116] Wu Y (2024). Research progress on the application of inkjet printing technology combined with hydrogels. Appl Mat Today.

[117] Li X (2020). Inkjet bioprinting of biomaterials. Chem Rev.

